# Network and co-expression analysis of airway smooth muscle cell transcriptome delineates potential gene signatures in asthma

**DOI:** 10.1038/s41598-021-93845-x

**Published:** 2021-07-13

**Authors:** Priyanka Banerjee, Premanand Balraj, Nilesh Sudhakar Ambhore, Sarah A. Wicher, Rodney D. Britt, Christina M. Pabelick, Y. S. Prakash, Venkatachalem Sathish

**Affiliations:** 1 Department of Pharmaceutical Sciences, North Dakota State University, Fargo, ND USA; 2 Department of Anesthesiology, Mayo Clinic College of Medicine, Rochester, MN USA; 3 Center for Perinatal Research, Abigail Wexner Research Institute at Nationwide Children’s Hospital, Columbus, OH USA; 4 Department of Pediatrics, The Ohio State University, Columbus, OH USA; 5 Department of Physiology and Biomedical Engineering, Mayo Clinic College of Medicine, Rochester, MN USA; 6 Department of Pharmaceutical Sciences, School of Pharmacy, College of Health Professions, North Dakota State University, Sudro 108A, Fargo, ND 58108-6050 USA

**Keywords:** Cell biology, Computational biology and bioinformatics, Respiratory tract diseases

## Abstract

Airway smooth muscle (ASM) is known for its role in asthma exacerbations characterized by acute bronchoconstriction and remodeling. The molecular mechanisms underlying multiple gene interactions regulating gene expression in asthma remain elusive. Herein, we explored the regulatory relationship between ASM genes to uncover the putative mechanism underlying asthma in humans. To this end, the gene expression from human ASM was measured with RNA-Seq in non-asthmatic and asthmatic groups. The gene network for the asthmatic and non-asthmatic group was constructed by prioritizing differentially expressed genes (DEGs) (121) and transcription factors (TFs) (116). Furthermore, we identified differentially connected or co-expressed genes in each group. The asthmatic group showed a loss of gene connectivity due to the rewiring of major regulators. Notably, TFs such as *ZNF792, SMAD1,* and *SMAD7* were differentially correlated in the asthmatic ASM. Additionally, the DEGs, TFs, and differentially connected genes over-represented in the pathways involved with herpes simplex virus infection, Hippo and TGF-β signaling, adherens junctions, gap junctions, and ferroptosis. The rewiring of major regulators unveiled in this study likely modulates the expression of gene-targets as an adaptive response to asthma. These multiple gene interactions pointed out novel targets and pathways for asthma exacerbations.

## Introduction

Asthma is a chronic inflammatory disease of airways characterized by inflammation, hyperresponsiveness (AHR), and remodeling^1–4^. Structural and inflammatory changes throughout the airway lead to bronchoconstriction contributing to airflow obstruction^5^. The airflow obstruction is due to a combination of increased smooth muscle in the airway (increase in number or size) and functional abnormality (increased contractility/decreased relaxation). This calls for attention to decipher the role of airway smooth muscle (ASM) cells in asthma exacerbations. ASM cells role in asthma pathophysiology is well elucidated^5–12^. Along with changes in airway structure and function, developmental abnormalities such as genetic disorders also play a role in asthma exacerbations^12^. Apart from being multifactorial, asthma is polygenic, and thus the transmission of disease through generations does not follow Mendelian inheritance^13^. Combinatorial action of genes and environmental factors results in altered gene expression patterns that could trigger the disease’s onset.

One of the approaches to assess the phenotypic differences between asthmatic and healthy states is by unraveling the genome-wide baseline gene expression profile in human ASM cells. Transformations in the asthmatic ASM cells physiological properties that could contribute to AHR would likely be manifested by deregulated gene expression patterns associated with the underlying biological pathway, which determines the overall functionality of cell/tissue. A plethora of genetic and genomic analysis^12–16^ and transcriptome analysis^17,18^ altering the molecular mechanisms associated with asthma are reported to discern the disease pathophysiology. Transcriptomic signatures with the response to glucocorticoid exposure on human ASM cells were reported previously^18^. In an independent study, *CRISPLD2* was identified as a glucocorticoid responsive gene modulating the cytokine function in human ASM cells^17^. Furthermore, the gene expression profile from laser microdissected ASM tissue in asthma showed, *RPTOR*, *VANGL1*, *FAM129A*, and *LEPREL1* were associated with AHR^19^.

Interestingly, all these studies identified the genes differentially expressed (DE) between asthmatic and non-asthmatic controls. In clinical research, altered or differential expression patterns can pinpoint the candidate biomarkers, therapeutic targets, and gene signatures for diagnostics. However, the particular gene expression pattern may not always translate into meaningful biological activity. Even though the differential expression may be involved with a biological pathway, it remains challenging to assess whether inhibition or activation of a specific biological pathway will alleviate or worsen the disease. Furthermore, the differential gene expression approach disregards the effect of multiple interactions involved with gene expression regulation^20^, a key target to phenotype determination. Additionally, transcription factors (TFs) play a pivotal role in the expression of smooth muscle contractile proteins and may modulate the phenotypes^21^. However, it is difficult to gain insight into gene regulation by TFs from differential expression analysis alone.

To overcome the limitations mentioned above, gene–gene co-expression networks are used to extract genome-scale information that encapsulates multiple regulatory systems’ activity. This approach has the potential to highlight specific molecular mechanisms in disease. Moreover, it focuses on a few highly connected hub genes and translates predictions into a testable hypothesis^22^. Gene co-expression networks are often constructed following guilt-by-association assumption representing nodes (genes) and edges (gene–gene associations)^23,24^, and this gene network topology provides a framework for molecular characterization^25^. To gain insights into the gene co-expression between various conditions of interest, differential co-expression analysis have emerged that operates on the level of gene-pairs rather than a single gene. Furthermore, a differential network analysis compares gene interconnections between groups (diseased and healthy), indicating differences in the underlying molecular mechanisms^26,27^ and identifying network rewiring in disease^28^.

Even though we have an insight into the molecular basis of asthma, no ASM cell-specific data is available on multiple gene–gene interactions that shed light on the molecular mechanisms involved with gene expression regulation. As a result, the extent of these differences affecting the coordinated function of genes and pathway regulation in asthma remains elusive. Therefore, we hypothesized that genes are differentially co-expressed (DCE), and gene networks are rewired in human asthmatic ASM cells. In the present study, we uncovered the interplay between the genes that are not only DE but also DCE and rewired between asthmatic and non-asthmatic human ASM cells. Herein, we identified regulatory interactions between the genes and network connectivity loss in the asthmatic group. This suggests a network rewiring of major regulators that modulates target genes' expression in response to the disease. Furthermore, on validation of pathway specific DEGs, we identified a significant fold change in the mRNA expression of genes quantified in human and mouse ASM.

## Results

### RNA-Seq transcriptome profiling of human ASM cells

**Figure 1:**
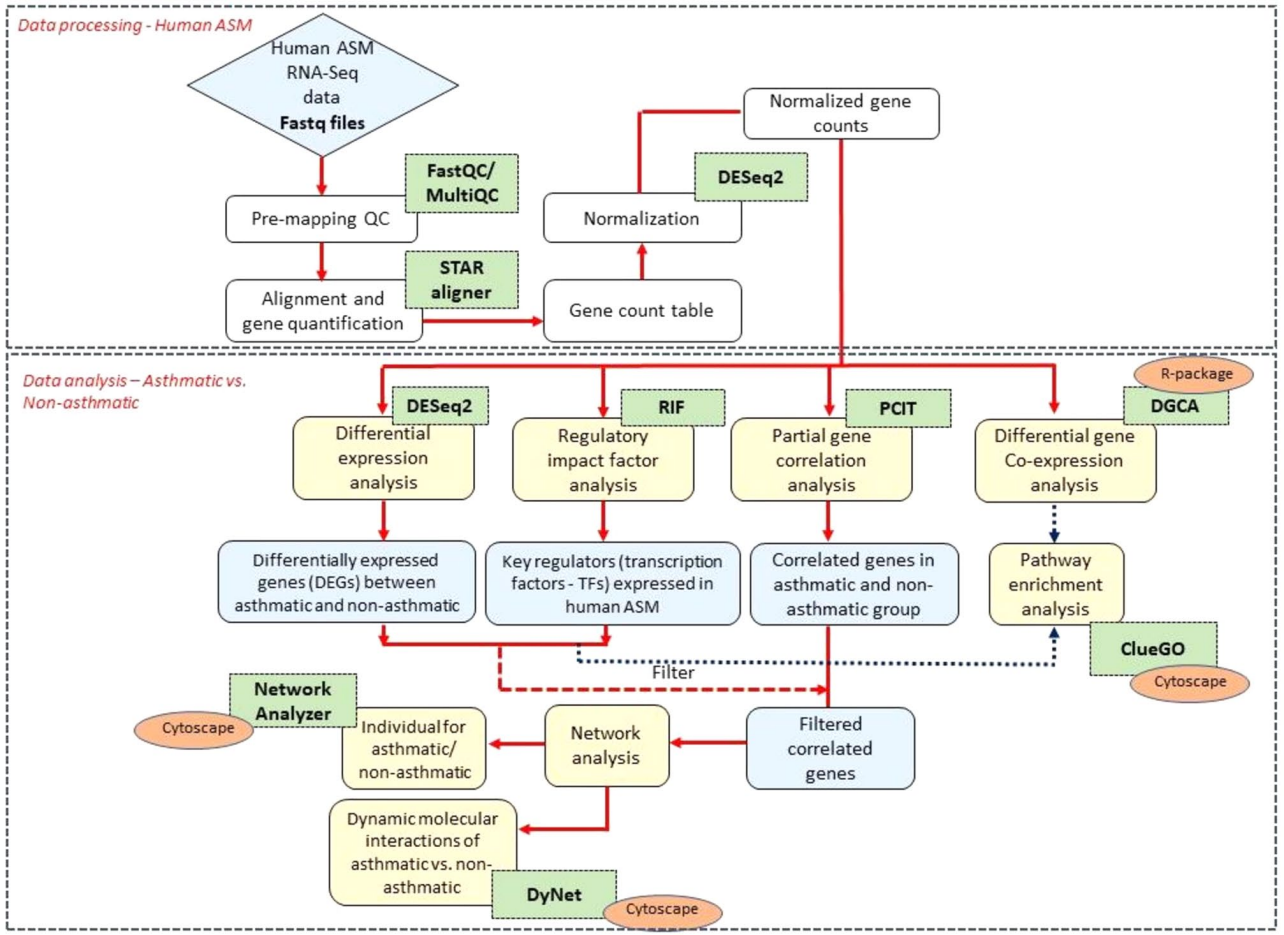
Schematic representation of the study design and analyses steps. Green boxes represent the software/packages used; yellow boxes are analysis steps.

**Figure 2:**
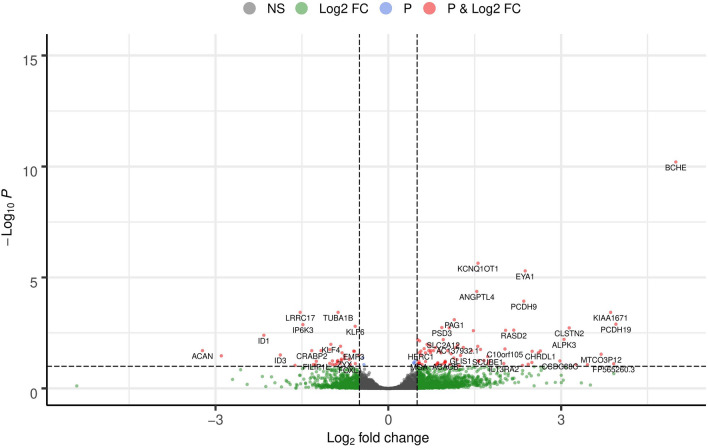
Volcano plot of gene-based differential expression analysis for asthmatics (case, N = 5) vs non-asthmatics (controls, N = 5) (each dot corresponds to a gene). The y-axis represents the negative log (base 10) of p-values, while the x-axis corresponds to the log (base 2) of the fold change for the difference in expression in the asthmatic human ASM cells. Pink dots represented 121 differentially expressed genes (padj < 0.1). The genes on the left of the panel (0 to − 3 of log2 fold change) are downregulated, while the genes to the right of the panel (0 to 5 of log fold change) are upregulated. Eighty-three genes were upregulated, while 38 genes were downregulated in the case group. The volcano plot was constructed using EnhancedVolcano v1.8.0 (https://doi.org/10.18129/B9.bioc.EnhancedVolcano) on R^97^.

The human ASM data from NCBI GEO GSE119578 were analyzed to ascertain the variation in the transcriptome profile of the asthmatic (case) vs. non-asthmatic (control). A schematic representation of the study design and analysis steps are given in Fig. 1. We obtained an average of 19.7 million raw sequencing reads per sample (ranging from 16.7 to 22.2 million reads per sample). The descriptive statistics and mapping rates per sample are reported in Supplementary Table S1. Of these samples, an average of 90.42% of reads was aligned to hg38 genome reference downloaded from the Ensembl database. Based on the QC summary metrics from FastQC and MultiQC, each sample’s sequencing and alignment results were above 90% and were included in the differential expression analysis. After the CPM filter approach, we identified 15,411 genes out of 60,671 reported in the Ensembl annotation file. We identified 121 genes significantly differentially expressed (DE) after correcting for false discovery rate by the Benjamini–Hochberg approach (Fig. 2, Supplementary Table S2). Among the DEGs, 83 genes were upregulated, such as *BCHE*, *PCDH19*, *PCDH9*, *KIAA1671*, *CLSTN2*, and the SLC family genes (*SLC2A12*, *SLC7A11*) (*padj* < 0.1) (Fig. 2). Thirty-eight genes were downregulated (Fig. 2) in the case, such as genes belonging to the tubulin family (*TUBA1B*, *TUBB6*, *TUBA1A*, *TUBA1C*), *LRRC17*, and *SLC39A14*. The top 10 significant genes upregulated and downregulated are given in Table 1. We also identified fifteen DE long non-coding RNAs (lncRNAs) such as *KCNQ1OT1, TMEM161B-AS1, GABPB1-AS1, AC137932.1, AC003681.1, MIR29B2CHG, Z95331.1, HOXB-AS2, AC103702.1, SAP30L-AS1, ZNF337-AS1, ARAP1-AS2, BX322234.1, LINC00886,* and *LOXL1-AS1* (*padj* < 0.1) relevant to asthma in our study (Supplementary Table S2).

**Table 1:** Differentially expressed genes identified (top 10) (upregulated/downregulated in asthmatics) from human airway smooth muscle cells.

Ensembl ID	Gene name	Status	Gene type	Chr no.	Gene start (bp)	Gene end (bp)	log2 FC	pvalue	padj
ENSG00000114200	*BCHE*	Up	Protein coding	3	165,772,904	165,837,462	4.99	4.07E−15	6.27E−11
ENSG00000269821	*KCNQ1OT1*	Up	lncRNA	11	2,608,328	2,699,994	1.56	2.96E−10	2.28E−06
ENSG00000104313	*EYA1*	Up	Protein coding	8	71,197,433	71,592,025	2.38	9.84E−10	5.06E−06
ENSG00000167772	*ANGPTL4*	Up	Protein coding	19	8,363,289	8,374,370	1.54	1.11E−08	4.27E−05
ENSG00000184226	*PCDH9*	Up	Protein coding	13	66,302,834	67,230,445	2.35	3.79E−08	1.17E−04
ENSG00000197077	*KIAA1671*	Up	Protein coding	22	24,952,730	25,197,448	3.86	1.81E−07	3.73E−04
ENSG00000076641	*PAG1*	Up	Protein coding	8	80,967,810	81,112,068	1.15	4.64E−07	7.95E−04
ENSG00000165194	*PCDH19*	Up	Protein coding	X	100,291,644	100,410,273	3.95	8.25E−07	1.27E−03
ENSG00000156011	*PSD3*	Up	Protein coding	8	18,527,303	19,084,730	0.93	1.52E−06	1.80E−03
ENSG00000158258	*CLSTN2*	Up	Protein coding	3	139,935,185	140,577,397	3.14	1.76E−06	1.87E−03
ENSG00000123416	*TUBA1B*	Down	Protein coding	12	49,127,782	49,131,397	− 0.87	1.51E−07	3.73E−04
ENSG00000128606	*LRRC17*	Down	Protein coding	7	102,913,000	102,945,111	− 1.53	1.94E−07	3.73E−04
ENSG00000161896	*IP6K3*	Down	Protein coding	6	33,721,662	33,746,905	− 1.48	9.51E−07	1.33E−03
ENSG00000067082	*KLF6*	Down	Protein coding	10	3,775,996	3,785,281	− 0.58	1.24E−06	1.59E−03
ENSG00000125968	*ID1*	Down	Protein coding	20	31,605,283	31,606,515	− 2.16	4.96E−06	4.02E−03
ENSG00000136826	*KLF4*	Down	Protein coding	9	107,484,852	107,490,482	− 1.00	1.60E−05	1.03E−02
ENSG00000183963	*SMTN*	Down	Protein coding	22	31,064,105	31,104,757	− 0.83	2.28E−05	1.26E−02
ENSG00000176014	*TUBB6*	Down	protein coding	18	12,307,669	12,344,320	− 1.02	4.11E−05	1.76E−02
ENSG00000157766	*ACAN*	Down	Protein coding	15	88,803,436	88,875,354	− 3.23	4.83E−05	1.98E−02
ENSG00000143320	*CRABP2*	Down	Protein coding	1	156,699,606	156,705,816	− 1.33	5.15E−05	1.98E−02

Differentially expressed genes were selected with padj < 0.1. The columns represent the Ensembl ID, Gene name, Status = Up/Downregulated, Gene type, Chromosome number, Gene start (bp), Gene end (bp), log2FC = log fold change, p-value, padj by Benjamini Hochberg criteria.

**Figure 3:**
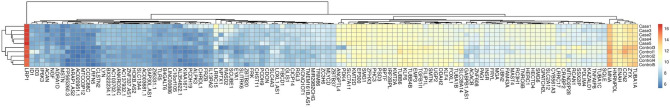
Heatmap and clustering analysis of differentially expressed genes (DEGs) and long non-coding RNAs (lncRNAs). The x-axis represents the 121 DEGs and lncRNAs; the y-axis represents asthmatic (case, N = 5), non-asthmatic (controls, N = 5). The case and control samples were grouped into two specific clusters, while the genes were grouped into two major cluster groups, including ten subgroups. The heatmap was constructed using pheatmap v1.0.12 (https://CRAN.R-project.org/package=pheatmap) on R^97^.

We observed the hierarchical clustering for DE genes and sample groups in the heatmap (Fig. 3). The case and control samples were grouped into two specific clusters (Fig. 3), while the genes were grouped into two major cluster groups, including ten subgroups.

### Regulatory transcription factors

TFs play a central regulatory role in controlling gene expression. We used regulatory impact factor (RIF1 and RIF2) analysis to assign a score and identify the TFs regulating the expression of DEGs. TFs with RIF1 and RIF2 z-scores less than − 2 or greater than 2 (threshold as suggested previously)^29^ were considered significant (Supplementary Table S3). We identified 116 TFs as the potential regulators modulating the expression of DEGs. *ZNF792* (z-score = 2.66) and *HOXB8* (z-score =  − 2.74) showed the most extreme value for RIF1 while *ZNF207* (z-score = 3.04) and *AFF3* (z-score =  − 2.82) were identified with the most extreme value of RIF2 (Supplementary Table S3). The genes encoding TFs are identified as key regulators and are reported as TFs throughout the text.

### PCIT and network analysis

We created two networks based on the partial correlation and information theory (PCIT) algorithm to identify differences in gene co-expression profiles between the conditions. The correlation of 15,411 genes retrieved 1,224,774 significantly correlated gene pairs in case, whereas 1,230,450 correlations for control. Herein, we aimed to identify the genes correlated with DEGs and TFs in our study. Thus, to identify the potential regulatory mechanism related to the disease status, we filtered the co-expressed gene pairs that were DEGs between case and control groups (121 genes), TFs based on RIF1 and RIF2 analysis (116 TFs), correlated pairs greater than |0.95|, for further analysis. After filtering, we retrieved 33,650 and 34,285 significantly correlated pairs in case and controls, respectively. These significantly correlated genes were used for the dynamic network analysis to visualize the most rewired nodes across the network from case and control.

To identify the case and control-specific connections, we analyzed each group’s networks separately using Cytoscape v3.8.0 (https://cytoscape.org/). For the case, after filtering out for DEGs and TFs, we identified 11,923 nodes and 33,650 edges, while 12,401 nodes and 34,825 edges for controls (Supplementary Table S4). Next, we determined the gene degree and selected the most connected genes. Genes with a high degree are hubs in the network and regulate important biological functions. We identified 236 hub genes in the case networks and 234 hub genes in control networks (p-value < 0.05). The top hub genes for the case networks are *SMAD7*, *ZNF180*, *ARNT*, *HIC1*, *TUBA1B*, *TUBA1C*, *TUBA1A* associated with more than 237 genes in the case. The top hub genes for control networks are *LRRC17*, *ZNF763*, *TUBB6*, and *CAPN15,* associated with more than 209 genes in controls. When overlapping the hub genes between conditions, *AFF3* and *ZNF718* are specific hubs in the case network (Supplementary Table S4).

**Figure 4:**
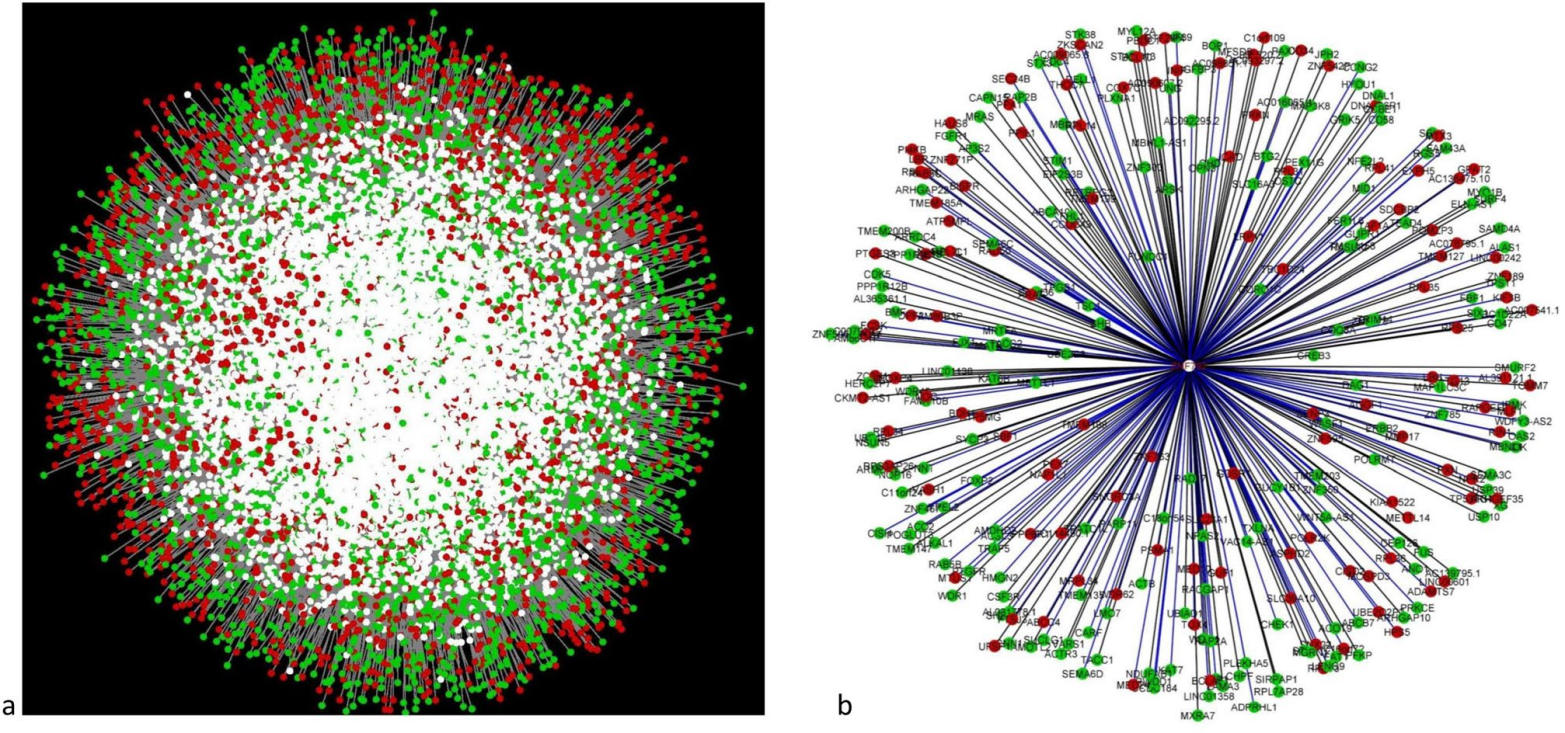
(**a**) Networks comparison based on rewired nodes. Central reference union network of genes co-expressed were built by DyNet merging case (N = 5) and control (N = 5) networks. The network comprises 14,720 nodes and 67,742 edges. Unique nodes are shown in green (control) and red (case). Shared nodes are shown in white. (**b**) Key regulatory transcription factor *ZNF792* and its co-expressed genes. The regulatory transcription factor is given in the middle with red font in white node; green nodes represent the target genes in the control network, whereas red nodes represent the target genes in the case network. Blue edges represent a positive correlation, while black edges represent a negative correlation between the transcription factor and the target genes. Gene network was created using Cytoscape v3.8.0 (https://cytoscape.org/).

The central reference network for case and control networks was constructed with DyNet that comprised 14,720 nodes and 67,742 edges (Fig. 4a, Supplementary Table S5). Based on differential connectivity (DK), we identified the most rewired genes between cases and controls. These networks followed a scale-free model with an R^2^ range of 0.65 in the case while 0.79 in the controls. We calculated DK between case and control, followed by a z-score for the 14,720 genes. For the z-scores, we considered 97.5 percentile points for standard normal distribution and retrieved the genes with z-score ± 1.96 (p-value < 0.05). By contrasting the connectivity between the groups, we identified 199 differentially connected (DC) genes for the case, among which 102 are DEGs and 97 are TFs. Among DC genes, 70 genes exhibited connectivity gain, whereas 129 genes lost connectivity due to asthma (Supplementary Table S5). The top DEGs that gained connectivity in the case are *TUBA1B*, *TUBA1C*, *TUBA1A*, *MAST4*, *TUBB6*, whereas the top TFs with connectivity gain in the case are *ZBTB14*, *ZNF461*, *AFF3*, *ZNF263*, *SMAD7*, *HOXB6*. Notably, *ZNF792* was earlier identified as regulatory TF based on extreme RIF scores exhibits connectivity loss in controls than in cases (Fig. 4b).

### Differential co-expression network analysis

We performed the differential gene co-expression analysis using DGCA R-package to reveal the functional relationship between gene pairs across the case and control groups on 15,411 genes. We identified 118,741,755 correlation pairs, out of which 3418 correlated pairs were significant (PValDiff_adj < 0.05, see Supplementary Table S6). Among them, 102 genes were DEGs or TFs.

Among the top 10 upregulated DEGs, *KIAA1671* was DCE with *PSMG3-AS1,* while *CLSTN2* was DCE with *BANF1* and *PMEL*. The top 10 downregulated DEGs, *LRRC17*, *SMTN*, and *ACAN,* were DCE with *CRADD*, *VAC14,* and *EPOP*, respectively. Interestingly, the co-expression of these genes was inversely correlated between case and control groups according to the correlation classes (see “Methods” section for details).

From the top hubs identified by Network Analyzer in the case group, TFs like *SMAD7* and *ARNT* were DCE with *WDCP* and *BTRC,* respectively. *TUBA1A* (DEG) was DCE with *RASAL2-AS1*, *SPON1*, *TF*, *AGAP9* with an inverse correlation between case and control groups. In control groups, the top hub genes *LRRC17* (DEG) and *CAPN15* (TF) were DCE with *CRADD* and *ADPRHL1,* respectively.

### Functional overrepresentation analysis

**Figure 5:**
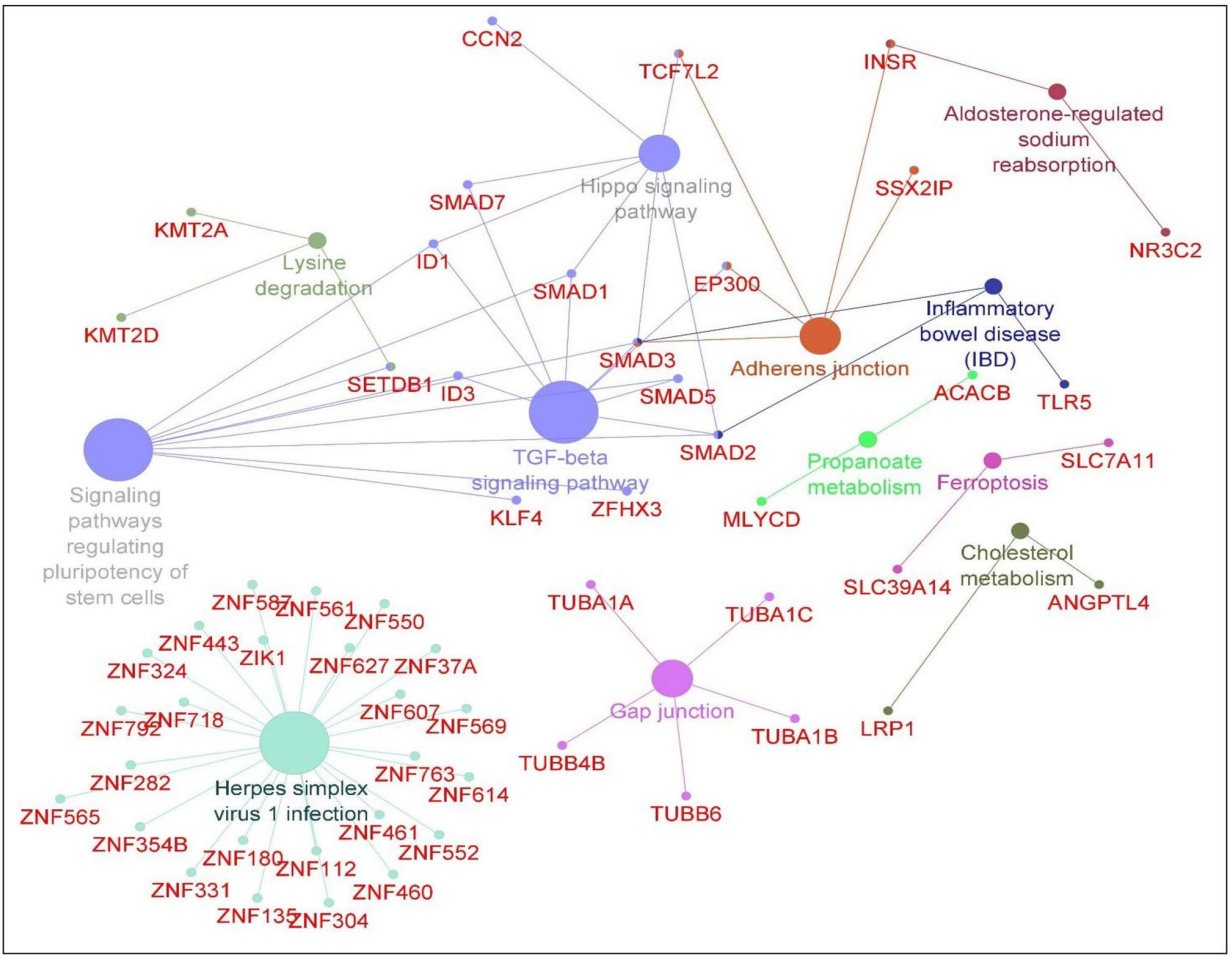
Over-represented signaling pathways of differentially expressed genes, transcription factors, and differentially connected genes. The circle size for a specific GOTerm (pathway) varies with the order of significance (p-value) level. The biggest node represents the most significant pathway. The pathways were constructed using Cytoscape v3.8.0 (https://cytoscape.org/) using the plugin ClueGO v2.5.4^105^.

To determine whether the biological functions or processes are over-represented by DEGs, TFs, and DC genes, we performed a network over-representation analysis. The top pathways identified were signaling like Hippo and TGF-β pathways, adherens and gap junction, cholesterol metabolism, ferroptosis, and lysine degradation aldosterone-regulated sodium reabsorption, and propanoate metabolism^30^ (p-value < 0.05, see Fig. 5, Supplementary Table S7). The pathways involved with DCE genes were included the Ras signaling pathway, phosphoinositide-3-kinase-Akt (PI3K-Akt) signaling pathway, mammalian target of rapamycin (mTOR) and mitogen-activated protein kinase (MAPK) signaling pathway, ferroptosis, cholesterol metabolism, adherens junction, Hippo and TGF-β signaling pathway (Supplementary Table S7). As ferroptosis was among the over-represented pathways, we screened the DEGs and TFs (237 genes) to the regulators of ferroptosis (253 genes) reported from the FerrdB database. We identified *SLC7A11*, *SLC2A12*, *ZEB1*, *ATF3,* and *HIC1* common between DEGs and TFs involved with asthma and ferroptosis.

### mRNA expression of identified genes in human and mouse ASM

**Figure 6:**
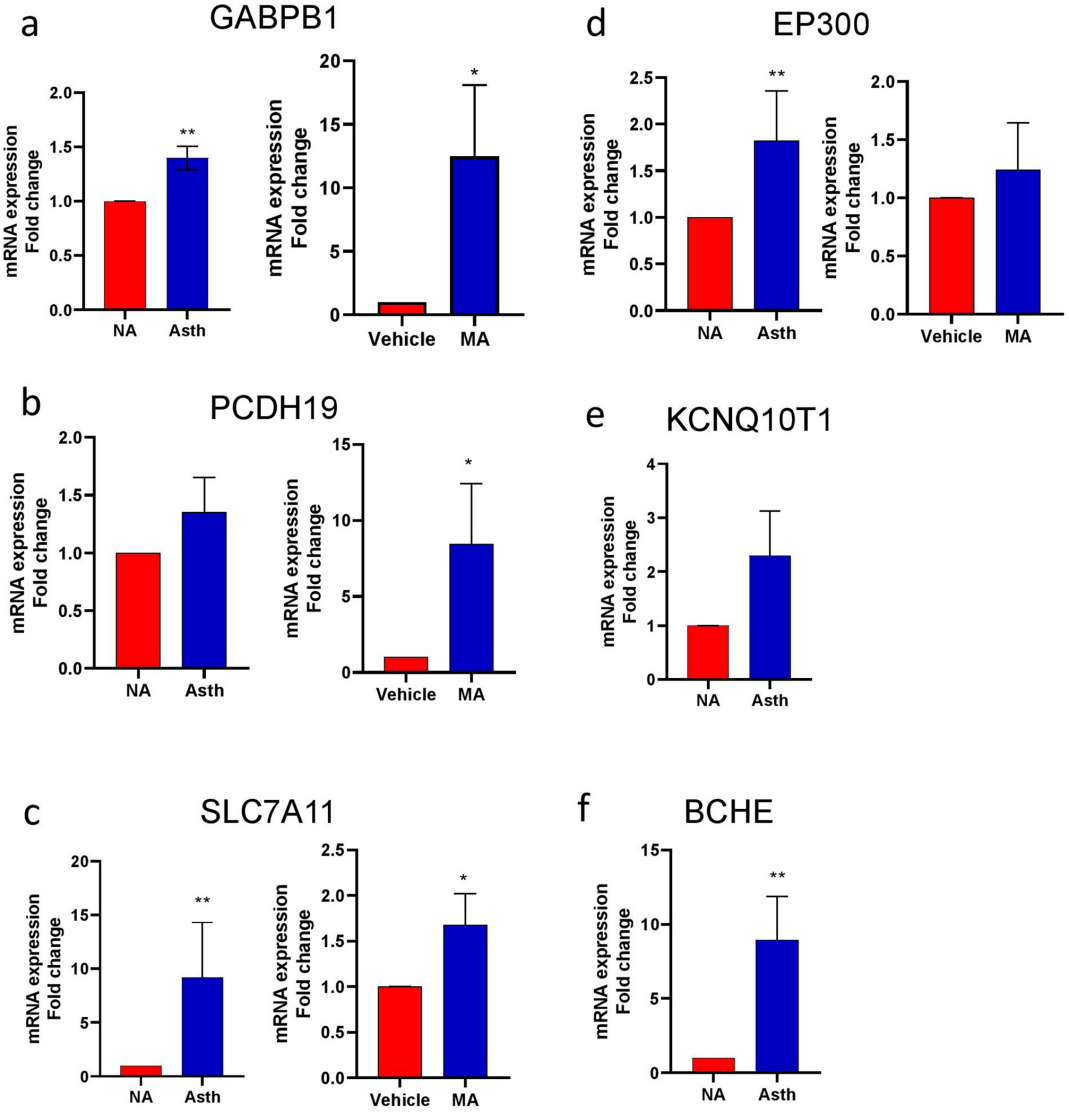
Differential mRNA expression of genes upregulated in human ASM and mouse ASM samples. The fold change for mRNA was evaluated in both groups. The genes were validated in human ASM samples collected from non-asthmatic (NA) and asthmatic (Asth) patients (N = 5 in each group) and mouse ASM samples collected with and without mixed allergen (MA) exposure (N = 3 in each group). Data represented as mean ± SEM in each group. *p-value < 0.05, **p-value < 0.001. The statistical analysis and the graphs were constructed using GraphPad Prism 9.0.0 (GraphPad, San Diego, CA) (https://www.graphpad.com/scientific-software/prism/).

**Figure 7:**
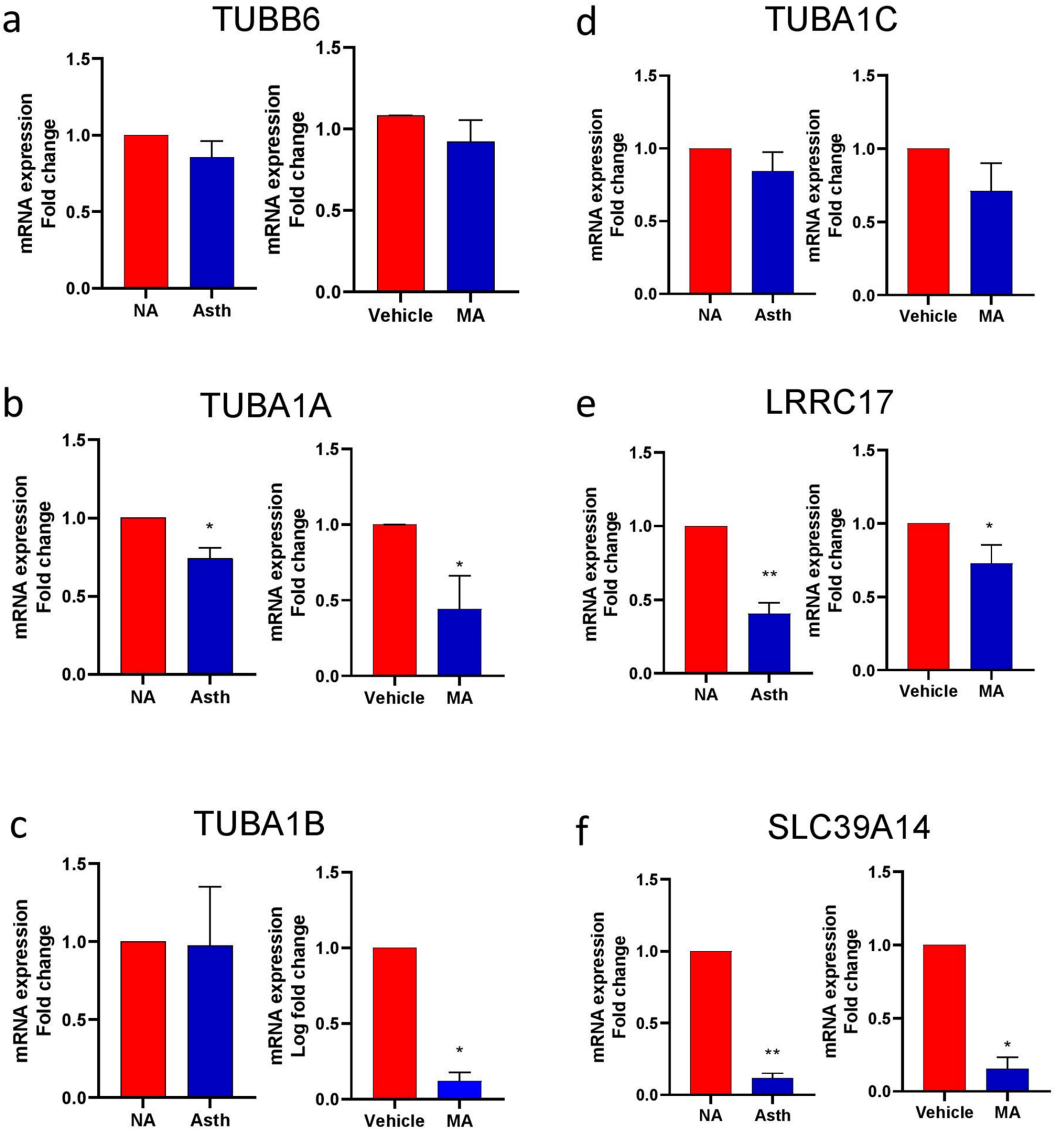
Differential mRNA expression of genes downregulated in human ASM and mouse ASM samples. The fold change for mRNA was evaluated in both groups. The genes were validated in human ASM samples collected from non-asthmatic (NA) and asthmatic (Asth) patients (N = 5 in each group) and mouse ASM samples collected with and without (MA) exposure (N = 3 in each group). Data represented as mean ± SEM in each group. *p-value < 0.05, **p-value < 0.001. The statistical analysis and the graphs were constructed using GraphPad Prism 9.0.0 (GraphPad, San Diego, CA) (https://www.graphpad.com/scientific-software/prism/).

Based on the results from identified DEGs and pathway-focused genes, multiple genes were selected to quantify whether gene expression changes could be observed in vitro and whether similar expression patterns extend to in vivo activity using the mixed allergen (MA) induced mouse model of asthma^3,31^. Consistent with the results mentioned above, *GABPB1*, *PCDH19*, *SLC7A11*, *EP300*, *KCNQ10T1,* and *BCHE* genes were identified as significantly upregulated in humans (p-value < 0.001) and mouse (p-value < 0.05) ASM cells (Fig. 6). Genes such as *TUBA1A*, *TUBA1B*, *TUBA1C*, *TUBB6*, *LRRC17*, *SLC39A14* were identified as significantly downregulated in human (p-value < 0.001) and mouse (p-value < 0.05) ASM cells (Fig. 7). Furthermore, RT-qPCR data showed an increase in mRNA expression for *ACTA2*, *CALD1*, *CNN1*, *MYH2* and *TPM1* genes in asthmatic ASM, but it was not statistically significant compared to non-asthmatic ASM, except in *TPM1*. No difference in the expression of *SMTN* was identified between asthmatic and non-asthmatic ASM cells, while *ACTG* was downregulated in the asthmatic ASM (Supplementary Fig. S1)*.* RNA samples extracted from the lung sections of PBS and MA exposed mice showed a significant increase in smooth muscle-specific marker expression (α-SMA) compared to the epithelial cells as a negative control. These data confirm the cells targeted and captured from LCM were precisely from smooth muscle and not from the epithelial layer.

## Discussion

ASM is well-known for its role in asthma exacerbations, characterized by bronchoconstriction and remodeling^7^. Since genes are co-regulated, unraveling the gene expression patterns across the whole genome in a healthy and diseased condition could reveal the novel gene signatures for asthma. Several studies reported DGE to discern the pathogenesis of asthma in children^32,33^ and adults^34,35^ involving the transcriptome profiling of airway epithelium^36–40^, bronchoalveolar lavage (BAL) cells^41^, and ASM cells^14,17,18^; however, no data is available on multiple gene–gene interactions that underlie the molecular mechanisms involved with gene expression regulation. Although DK of gene regulatory networks in asthma is reported in immortalized B cells following corticosteroid treatment^42^, to our knowledge, there are no studies on human ASM cells. As a result, the extent of these differences between normal and diseased conditions affecting the coordinated function of genes and pathway regulation in asthma remains elusive. To overcome this limitation, we used gene co-expression and DK of gene networks to shed light on the biologically related functions underlying asthma.

Herein, we identified 121 DEGs and 15 lncRNAs in human ASM cells between case and control groups. Additionally, based on the RIF approach, we identified 116 TFs acting as putative DEGs regulators. TFs bind to transcription factor binding sites (TFBS), thereby playing a role in transcription regulation^43^. A better understanding of the TFs regulating gene expression will help us to understand the transcriptional regulation mechanism.

To provide an overview of gene expression relationships in both case and control groups, we prioritized genes from DGE and TF analyses to identify specific co-expression differences between the groups. This was done using DGCA and DK analyses to identify hub genes with significant biological roles. Additionally, we found a loss of connectivity in the ASM gene network from the case group. This suggests a network rewiring of major regulators that modulates target genes’ expression in response to the disease.

The over-representation analyses of DEGs, TFs, DC, and DCE genes identified pathways involved with herpes simplex infection, Hippo signaling pathway, TGF-β signaling pathway, adherens junction, gap junction, cholesterol metabolism, lysine degradation, ferroptosis, and aldosterone-regulated sodium reabsorption and propanoate metabolism.

LncRNAs function as modulators in asthma pathophysiology^44,45^, particularly in ASM cells^46,47^. In our study, we identified 15 DE lncRNAs along with differentially expressed genes in ASM cells. Among them, *KCNQ10T1* was among the top 10 DE lncRNA and more connected in controls (138) than in the case (97). Moreover, the RT-qPCR results exhibited an increase in the mRNA expression in the case as compared to the control group. LncRNA *KCNQ10T1* was significantly different in the serum of asthmatic children with airway remodeling and those without remodeling^48^. Moreover, *KCNQ10T1* was reported to be one of the significant predictors of airway remodeling in children with bronchial asthma^48^. *GABPB1-AS1* (upregulated in case) was DE and DK with a connectivity gain*.* The protein-coding gene (*GABPB1*) underlying *GABPB1-AS1* also exhibited an increase in mRNA expression in asthmatic human ASM cells and MA exposed mouse ASM cells. *GABPB1-AS1* regulates oxidative stress in erastin induced ferroptosis^49^. Qi et al*.* reported that erastin upregulated the lncRNA *GABPB1*-*AS1*, which in turn downregulates *GABPB1* protein (blocks its translation) and *PRDX5* (peroxiredoxin-5 peroxidase), thereby suppressing antioxidant capacity^49^. Notably, previous studies reported aberrations in oxidant: antioxidant balance can contribute to asthma^50^. Overall, these studies suggest a relationship between ferroptosis and asthma, indicating *GABPB1-AS1* as a potential lncRNA target to be further investigated.

We identified two upregulated lncRNAs, *AC137932.1* and *ARAP1-AS2,* that are antisense to an ankyrin gene family with *ANKRD11* and *ARAP1* gene, respectively. Briefly, ankyrins are a group of adaptor proteins that link the integral submembranous actin/β-spectrin cytoskeleton^51^. They have a pivotal role in the formation of protein complexes consisting of ion channels and transporters, cell adhesion molecules, signaling proteins, and cytoskeletal elements^51^. Specifically, the interplay between the *ANKRD11* gene with asthma is still not reported; however, *ANKRD11* has a role in the proliferation and development of cortical neural precursors^52^. The neurophenotypes for asthma have been linked previously by brain circuits’ neural reactivity to be involved in processing emotional information^53^. *ARAP1* gene encodes for a protein that displays RHO-GAP (GTPase activating protein) and (PIP3)-dependent ARF-GAP activity that controls the trafficking and signaling of EGF-R (epidermal growth factor receptor)^54^. Additionally, Rho-kinase attenuates airway responsiveness, inflammation, remodeling, and oxidative stress induced by chronic inflammation^7,55^.

A subset of homeobox genes, members of the *HOX* gene family, were identified as DE and DC in asthma. *HOXB* cluster genes comprising *HOXB-AS2* and *AC103702.1* (antisense to the intron of *HOXB3* gene) are the upregulated lncRNA, DC with a connectivity gain in the case group in the present study. Herein, *AC103702.1* is DCE with *AC010896.1* (+/−). *HOXB3* is known to be important in lung development^56^ and is an upstream regulator of thyroid transcription factor-1 (TTF-1) expressed in developing thyroid, lung, and brain. Inhibition of TTF-1 during lung morphogenesis in vitro disrupts normal differentiation of lung epithelial cells^56^. Apart from the lncRNAs discussed above, other lncRNAs DE in our study, such as *SAP30L-AS1* and *BX322234.1,* showed increased connectivity for the case groups. *ZNF337-AS1* was DCE with *PPCDC* (−/+), *EID2* (+/−), and *PIAS3* (−/0) in our study. These findings suggest that lncRNAs have a potential role in asthma regulation and that the novel targets identified could be explored for asthma exacerbations.

The genes differentially expressed in asthmatic human ASM also over-represented for Herpes simplex virus infection pathway. Herpes simplex virus type 1 (double-stranded DNA virus) can cause acute or latent infections in humans. Impaired immune response in asthma increases the susceptibility to herpes infections. T-cell activation and skewed Th1/Th2 are critical to the initiation and maintenance of airway inflammation in patients with asthma. Moreover, an enhanced Th2 immune response and elaboration of cytokines are associated with asthma^57^. The herpes simplex virus infection is predominantly associated with T-cell mediated immunity^58^. Herpes patients have a low Th1 response and a high Th2 response compared to control^59^. Thus, Th1 immune deficiency in asthma could increase their susceptibility to herpes simplex infections. Our study identified DE TFs such as *ZNF180*, *ZNF763*, *ZNF792*, *ZNF718*, and *ZNF461* associated with asthmatic ASM cells and enriched for the herpes simplex infection pathway. While *ZNF180*, *ZNF718*, and *ZNF461* showed increased connectivity in the case group, *ZNF763* and *ZNF792* lost connectivity. Here, *ZNF792* had the highest RIF1 score and was identified as the potential regulators of DEGs. Several TFs are known to be relevant to asthma, including glucocorticoid receptor (GR), nuclear factor of activated T-cells (NF-AT), cyclic-AMP response element-binding protein, peroxisome proliferator-activated receptor (PPAR), etc.^60^; however, to the best of our knowledge, the role of the zinc-finger family have still not been explored. To advance the understanding of asthma, their inhibition/activation at the expression level or at the protein/DNA binding level still requires investigation, specifically with human ASM.

The DEGs (*EP300*, *ID1*, *ID3*) and TFs (*SMAD1*, *SMAD2*, *SMAD3*, *SMAD5,* and *SMAD7*) expressed in human ASM cells were involved with the TGF-β signaling pathway. In our study, *EP300*, *ID1*, *ID3*, *SMAD2,* and *SMAD5* were DC with a loss of connectivity, whereas *SMAD7* exhibited a network connectivity gain due to asthma. Among the genes DCE in our study, we identified *ID3* with *AC083862.2* (−/+), *SMAD1* with *AC012513.3* (−/+), and *PLEKHN1* (0/−), and *SMAD7* with *WDCP* (−/+), respectively. In a study reported by Gunawardhana et al., the expression of EP300 was not altered in asthmatic patients vs. healthy controls^61^. On the contrary, *EP300* was upregulated in our study. The fold change from mRNA expression through RT-qPCR validation also exhibited an upregulation of *EP300* in the asthmatic group. The genes identified as mentioned above were involved with TGF-β signaling. TGF-β can induce cellular responses like differentiation and proliferation and have been implicated in asthma development^62^. TGF-β activates multiple pathways involving proteins, such as SMAD, leading to the transcription of several genes. The effect of the SMAD pathway is observed to have both stimulatory and inhibitory effects^62^. As a stimulatory effect, SMAD proteins interact with TGF-β receptors through a conserved MAD-homology-2-domain^62^. TGF-β activates the SMAD pathway by phosphorylation of *SMAD2/3* by a subsequent translocation into the nucleus. On the other hand, the inhibitory effects of SMAD involve *SMAD7* blocks transcription of mRNA and inhibit the pathway, thereby preventing the phosphorylation of *SMAD2/3* and further downstream signaling^62^. The role of TGF-β/SMAD signaling is well associated with airway remodeling in asthma^63–66^. The SMAD gene identified is also enriched in the Hippo signaling pathway in the present study. The hippo signaling pathway plays a crucial role in growth control, proliferation, and tumor suppression by coordinating the cellular processes through crosstalk with TGF-β^67^.

The genes regulating cytoskeleton were identified in human ASM regulating asthma. The cytoskeleton is composed of a variety of structural and contractile proteins responsible for maintaining cell morphology. The cytoskeleton exists in three main forms: microfilaments, microtubules, and intermediate filaments^68^. Microtubules form cytoplasmic networks regulating cellular shape, intracellular organization, axonal flow, and chromosomal segregation^69^. Tubulin is one of the major proteins of microtubules^70^ that regulates smooth muscle cell migration and airway remodeling^71^. In the present study, we identified tubulin family genes DE and related to asthma. We identified *TUBA1A*, *TUBA1B*, *TUBA1C*, *TUBB4B,* and *TUBB6* downregulated in the case, while the genes exhibited a connectivity gain with the disease. Interestingly, *TUBA1A* was identified to be DCE with *AGAP9* (−/+) and *RASAL2-AS1* (+/−); *SPON1* (−/+); and transferrin (*TF*) gene (−/+). *TUBA1A* was associated with glycolysis, calcium biding, and proteomic analysis in early asthmatic response in rats^72^. Moreover, another study pointed out that the expression of α-tubulin is inhibited by NK1R (neurokinin-1 receptor) antagonists that suppress ASM cell migration in rats^73^. In addition to the few studies of tubulin in asthma, the role of tubulin in the nervous system and neurodegenerative diseases such as Parkinson’s and Alzheimer’s are well known^74^. An independent study reported that asthma was associated with an increased risk of developing dementia and Alzheimer’s disease^75^. On validation of *TUBA1A*, *TUBA1B*, *TUBA1C,* and *TUBB6* in human and mouse ASM through RT-qPCR, we identified a decrease in *TUBA1A TUBA1B* mRNA expression levels*.* Interestingly, in this study, tubulin is downregulated; however, the other microtubule-related genes up or down-regulated during diseased conditions might play a major role during the ASM proliferation and migration process. Further validation of the targets and related pathways identified in the present study could be a possible link to decipher the role of tubulin in asthma exacerbations.

We identified *SLC7A11* and *SLC39A14* genes DE in ASM cells and over-represented for ferroptosis in the present study. Ferroptosis is programmed cell death characterized by an excessive accumulation of iron and lipid peroxides. Several studies have reported a close association of ferroptosis with the respiratory disease^76^, tuberculosis^77^, chronic obstructive pulmonary disease (COPD)^78^, and asthma^79^. Both genes identified in this study have more connectivity in controls than in the case, which implies that they lose their connectivity in asthma. The relative fold change from mRNA expression showed similar expression patterns in *SLC7A11* (increase) and *SLC39A14 (decrease)* in vivo and in vitro studies. In a previous study, *SLC7A11* was identified as a ferroptosis-related gene in airway epithelial cells that predicted asthma exacerbations^80^. From the DGCA analysis, we identified *ACSL3*, *ACSL5*, *FTL*, *GCLC*, *GCLM*, *MAP1LC3B2*, *MAP1LC3C*, *PCBP2*, *SLC11A2*, *STEAP3*, *TF,* and *TFRC* DCE and over-represented for ferroptosis. Previous studies reported the role of glutamate-cysteine antiport system Xcd^−^ comprising of a catalytic subunit *SLC7A11* in ferroptosis^81^. System Xc^−^ mediates the exchange of cystine (converted to cysteine and imported in intracellular space), and glutamate (exported to extracellular space) is crucial for glutathione (GSH) synthesis^82^. GSH protects cells from oxidative damage by reducing reactive oxygen species (ROS). ROS, in turn, modulates mitochondrial structure and function, thereby promoting oxidative stress, airway inflammation, and pathogenesis^83^. Oxidative stress characterized by lipid peroxidation is believed to contribute to asthma pathophysiology^84^ and also plays a crucial role in triggering ferroptosis^85^. From the genes, DCE, *ACSL3,* and *ACSL5* are expressed in mitochondrial outer membrane catalyzes fatty acids to form acyl-CoA^85^. Moreover, *ACSL3*-dependent MUFA (monounsaturated fatty acids) metabolism is a crucial regulator of ferroptosis cell death^86^. While *ACSL3* is associated with asthma susceptibility^87^, its role in ferroptosis and asthma still needs to be investigated. Several other factors regulate ferroptosis, such as the antioxidant enzyme: *GPX4* and the TF Nrf2. *GPX4* is a regulator of ferroptosis by opposing lipid peroxidation, while Nrf2 plays an important role in iron and lipid metabolism^76^. The transcription targets of Nrf2 such as *GCLC/GCLM* (GSH catalysis and modulation dependent subunits), *GSS* (glutathione synthetase), and *SLC7A11* were DCE in our study. Other DCE genes for asthma over-represented for ferroptosis included transferrin and *STEAP3.* Transferrin is a circulating glycoprotein that binds to the ferric iron mainly converted from dietary iron. Ferric iron is transformed to ferrous iron through *STEAP3* (six transmembrane epithelial antigens of the prostate 3)^76^. Thus, all the proteins aforementioned for a gene-targeted regulation of iron can mediate ferroptosis by modulating the level of intracellular iron. While all these genes were identified as a DE and DCE in asthma, highlighting their intricate role in ferroptosis, however, the basic molecular mechanisms of ferroptosis in asthma remain in their infancy. The genes identified for ferroptosis in a diseased condition may contribute to increase or decrease in ASM mass in asthma-induced remodeling, which warrants further detailed studies.

## Conclusions

The current study demonstrates differences in the ASM gene expression profiles associated with asthma. Furthermore, we identified and validated putative major regulators driving the gene-expression and reported differences in the regulatory mechanisms involved with asthmatic ASM. Expression of the genes, regulatory TFs, and the underlying pathways identified in this study regulating human ASM transcriptome provide new targets for asthma management. This research’s originality lies in the multiple approaches used in determining gene–gene interactions during asthma. A detailed understanding of these genes, TFs, and pathways in asthmatic ASM cells/tissues could lead to new therapies to prevent asthma exacerbations.

## Methods

### Data resource and phenotype collection

This study was conducted on an RNA-Seq dataset from human ASM cells (NCBI GEO GSE119578). The experimental design, sample collection, and library preparation for RNA-Seq are previously reported^88^. For human samples, written informed consent was obtained from all subjects, and the study was approved by the Mayo Clinic Institutional Review Board. All methods were performed in accordance with relevant guidelines and regulations. In brief, human ASM cells were isolated at the Mayo clinic from non-asthmatic and asthmatic patients with the previously described procedures^6,89,90^. Pathologically normal area of third-to-sixth-generation human bronchi from lung specimens was collected. The epithelium was removed, and the remaining ASM tissue was enzymatically dissociated as previously described^91^. Cells were grown in DMEM/F12 media supplemented with 10% fetal bovine serum (FBS) and 1% antibiotic/antimycotic (AbAm). After the cells reached 80% confluency, they were serum-starved with DMEM/F12 media (without FBS) and 1% AbAm for 48 h. RNA isolation was performed from cell lysates at Mayo clinic, as reported elsewhere^88^. After RNA quality control, RNA libraries were prepared using 200 ng total RNA per the manufacturer’s instructions for TruSeq RNA Sample Prep Kit v2 (Illumina). Sequencing was carried out on Illumina HiSeq 2000 platform. The sequence generated were paired-end (51-bp reads) with ~ 30–45 million reads per sample.

### RNA-Seq, differential expression, and statistical analyses

The RNA-Seq data from human ASM (10 independent samples) was processed and analyzed using the *Center for computationally assisted science and technology* (CCAST) resources (https://www.ndsu.edu/it/help/ccast/) at NDSU. Data quality control (QC) was performed using FastQC v0.11.8^92^ and MultiQC v1.9^93^. The reads were mapped using STAR aligner^94^ v2.7.5a using human genome reference (hg38) and gene annotation (Homo_sapiens.GRCh38.gtf) from the Ensembl database. The read quantification was done using -*quantMode* gene counts flag to obtain the raw counts per gene. Read counts were transformed to counts per million (CPM) using edgeR, and genes with low count (expression) with CPM < 0.5 in 50% of the samples were filtered out. Post-mapping QC was performed using MultiQC and edgeR^95^. The differential gene expression (DGE) analysis and normalization of the filtered gene counts were done using DESeq2^96^. Due to the contribution of covariates to the total gene expression, the age and gender of the samples were considered for the design model of DESeq2. Differentially expressed genes (DEGs) were selected with *padj* ≤ 0.1. The genes with log-fold chain—LFC > 0 were considered upregulated, while LFC < 0 were considered downregulated. The upregulated and downregulated genes were visualized using the volcano plot constructed using EnhancedVolcano v1.8.0 (https://doi.org/10.18129/B9.bioc.EnhancedVolcano) on R^97^ The normalization for the filtered genes (n = 15,411) were performed by variance stabilizing transformation (VST) function in DESeq2^96^. Cluster analysis and heatmap of DEGs were generated using the ‘pheatmap’ package (v1.0.12) (https://CRAN.R-project.org/package=pheatmap) in R. The asthmatic and non-asthmatic groups will be referred to as case and control groups in the manuscript.

### Identification of key regulators

Regulatory impact factor (RIF) metrics were used to identify the putative TF differentially regulating gene expression profiles between the groups in human ASM cells. RIF analysis identified the regulators of differential expression in two biological conditions. While RIF1 identifies TFs that are consistently most differentially co-expressed with highly abundant and DEGs, RIF2 identifies those TFs with the most altered ability to predict the abundance of DEGs^29^. TFs were downloaded from the human transcription factor database (Human TFDB v3.0)^98^ and contrasted to the list of DEGs identified, comparing case vs. control.

### Co-expression profile, filtering with DEG and TFs, and gene networks

To identify the gene co-expressed by correlation analysis, PCIT (Partial correlation and information theory) algorithm was used for the normalized data^99^. PCIT compares all possible triplets of genes by exploring the concepts of partial correlation and mutual information, thereby reporting the significantly correlated pair after screening all the genes in the network^99^. Significant gene–gene correlations were considered based on the p-value < 0.05, r >|0.95|, and included DEGs and TFs. The case and control co-expression networks were constructed using PCIT. The networks were analyzed using the Network Analyzer tool^100^ in Cytoscape v3.8.0, and the hub genes were identified using the degree measure considering the Mean + 2SD. The networks were analyzed for the degree of distributions. The degree measure was used to ascertain the highly connected genes (hubs) by considering the Mean + 2SD (standard deviation) of the degree identified from the Network analyzer tool. In the groups, the visualization of nodes and edge rewiring within the molecular interaction networks was done using a Cytoscape plug-in DyNet^101^. DyNet allows for identifying and visualizing connectivity changes in response to cellular signals and highlights the most rewired nodes based on a central reference network constructed from case and control groups^101^.

To identify the differentially connected (DC) genes in each group, each network’s connectivity (K) measures were standardized by taking a ratio of gene connectivity and maximum connectivity^102^. The differential connectivity (DK) measure was calculated as $DKi = KCase\left( i \right){-}KControl\left( i \right).$ The DK values were transformed into a z-score, and ± 1.96 SD was considered significant (p-value < 0.05). The networks were visualized in Cytoscape v3.8.0^103^ (https://cytoscape.org/). For the differential co-expression analysis between the groups, R-package DGCA version 1.0.2 was used^104^ on filtered and normalized gene counts (n = 15,411). With DGCA, the sample correlation coefficients variance in case and control groups were stabilized by the z-score transformation. For multiple hypothesis testing corrections, p-values were adjusted using the Benjamini–Hochberg method (*padj* ≤ 0.05). The gene-level pairs were classified as having a gain of correlation or loss of correlation, and based upon the threshold for correlation significance; the DK gene pairs were grouped into nine different correlation classes (+/+; +/−; +/0; −/+; −/0; −/−; 0/+; 0/0; 0/−). The classes show correlation as positive (+), negative (−), or not significant (0) for each gene and condition when contrasting the groups (case/control).

### Pathway analysis and comparison with FerrdB

The pathway over-representation analysis for DEGs, TFs, DC, and DCE was done using ClueGO v2.5.4 (Cytoscape plug-in)^105^ (https://cytoscape.org/). The annotation of the Ensembl IDs for the respective genes was done using BiomaRt (R package)^106,107^ from the human Ensembl database, *GRCh38.p13*. The pathways over-represented in the selected modules were identified by grouping the redundant terms with kappa-score = 0.4. Pathways with a p-value < 0.05 were considered significant. The identified DEGs and TFs were compared with FerrDb—a manually curated resource for regulators and markers of ferroptosis—to identify the common regulators between asthma and ferroptosis.

### RT-qPCR analysis for gene expression in ASM cells from human and mouse lung tissue

The significant genes up/downregulated and involved in the top pathways were validated in human and mouse ASM cells. Human ASM cells were washed with DPBS (Dulbecco’s phosphate buffer saline), trypsinized, and centrifuged. The total RNA was extracted using the Quick-RNA Miniprep Kit (Zymo Research, Irvine, CA) following the manufacturer’s protocol.

Mouse ASM cells were collected from the lung tissue sections of the vehicle (PBS), and mixed allergen (MA) exposed C57BL/6J mice following the previous protocols^108^. In brief, murine lung tissue samples were collected from an ongoing pulmonary in vivo study, approved by the Institutional Animal Care and Use Committee (IACUC) at NDSU following the National Institute of Health (NIH) guide for the care and use of laboratory animals. Animals were anesthetized with isoflurane for 10 s and then treated intranasally with PBS or MA on alternate days for four weeks. The MA exposure regimen included equal amounts (10 µg each) of ovalbumin, extracts from *Alternaria alternata*, *Aspergillus fumigatus,* and *Dermatophagoides farina* (house dust mites). The mice were housed at constant temperature and 12 h light and dark cycles, provided with food and water ad libitum. The lung tissue was collected from the mouse and fixed with paraformaldehyde for extraction of mouse ASM cells. The tissue blocks were embedded in paraffin wax, sectioned to 10 µM thickness, and processed as described elsewhere^108^. The clean slides were subjected to laser microdissection (LCM) using a Zeiss Axio Imager Z1 PALM MicroBeam laser capture system (Zeiss, Thornwood, NY). After identifying airways from the lung section on the slide^108^, the ASM tissues were collected into the cap of an RNAse free microtube containing a 0.75 ml single-cell PicoPure RNA extraction buffer. The cells were stored at − 80 °C or directly used for RNA isolation. The total RNA was extracted using PicoPure RNA Isolation Kit (Thermo Fisher Scientific, Waltham, MA, USA) following the manufacturer’s protocol.

RNA concentration and purity were assessed using Synergy HTX (Biotek, USA). Sample absorbance was measured at 260 nm and 280 nm, and the 260/280 ratio was used to assess the RNA purity. The RNA purity was considered adequate when the 260/280 ratio was ≥ 2.0. The total RNA extracted from human and mouse ASM was reverse transcribed using a OneScript Plus cDNA Synthesis Kit (Applied Biological Materials Inc, Richmond, BC, Canada). The cDNAs were subjected to RT-qPCR using BrightGreen 2× qPCR MasterMix reagents (Applied Biological Materials, Canada). The primer sequences used for the RT-qPCR analysis of cDNA prepared from human and mouse ASM are given in Supplementary Table S8. The fold change in mRNA expression was calculated using the ΔΔCt method by normalizing the cycle threshold value of the targeted gene of interest to housekeeping genes s16 included in each plate^109^. Furthermore, the human ASM from asthmatic and non-asthmatic groups were evaluated for smooth muscle-specific markers such as *ACTA2*, *ACTG*, *MYH2*, *TPM1*, *CNN1*, *SMTN* and *CALD1*. To confirm the purity of smooth muscle samples collected from mouse airways by LCM, the fold change expression of smooth muscle-specific marker such as α-smooth muscle actin (α-SMA) was evaluated against the mouse epithelial cells (as negative control). The statistical analysis and the graphs were constructed using GraphPad Prism 9.0.0 (GraphPad, San Diego, CA) (https://www.graphpad.com/scientific-software/prism/), and the results are reported as mean ± SEM. Statistical analysis was performed using the Mann–Whitney test (non-parametric), and statistical significance was tested at a p-value < 0.05.

## Supplementary Information

Supplementary Tables.

Supplementary Figures.

## Data Availability

The RNA-Seq data for human ASM (10 independent samples) were submitted to NCBI: GEO GSE119578.

## Abbreviations

AHR: Airway hyperresponsiveness
ASM: Airway smooth muscle
BAL: Bronchoalveolar lavage
DC: Differentially connected
DCE: Differentially co-expressed
DE: Differentially expressed
DEGs: Differentially expressed genes
DK: Differential connectivity
GEO: Gene expression omnibus
lncRNAs: Long non-coding RNAs
NCBI: National Center for Biotechnology Information
PCIT: Partial correlation and information theory
RIF: Regulatory impact factor
RT-qPCR: Real-time quantitative polymerase chain reaction
TFBS: Transcription factor binding sites
TFs: Transcription factors
