# Supplementary material for: Network and co-expression analysis of airway smooth muscle cell transcriptome delineates potential gene signatures in asthma
Source: Sci Rep. 2021 Jul 13;11:14386. doi: 10.1038/s41598-021-93845-x (PMC8277837; doi:10.1038/s41598-021-93845-x)
Supplement: Supplementary file 2 — Supplementary Figures. [file 41598_2021_93845_MOESM2_ESM.pdf]

Network and co-expression analysis of airway smooth muscle cell transcriptome delineates potential gene signatures in asthma

Priyanka Banerjee[1], Premanand Balraj[1], Nilesh Sudhakar Ambhore[1], Sarah A Wicher[2,] Rodney D Britt Jr.[3,4], Christina M Pabelick [2,5], Y. S. Prakash [2,5], Venkatachalem Sathish[1]*

[1]Department of Pharmaceutical Sciences, North Dakota State University, Fargo, North Dakota
[2]Department of Anesthesiology, Mayo Clinic College of Medicine, Rochester, Minnesota
[3]Center for Perinatal Research, Abigail Wexner Research Institute at Nationwide Children's Hospital, Columbus, Ohio
[4]Department of Pediatrics, The Ohio State University, Columbus, Ohio
[5]Department of Physiology and Biomedical Engineering, Mayo Clinic College of Medicine, Rochester, Minnesota

SUPPLEMENTARY FIGURE

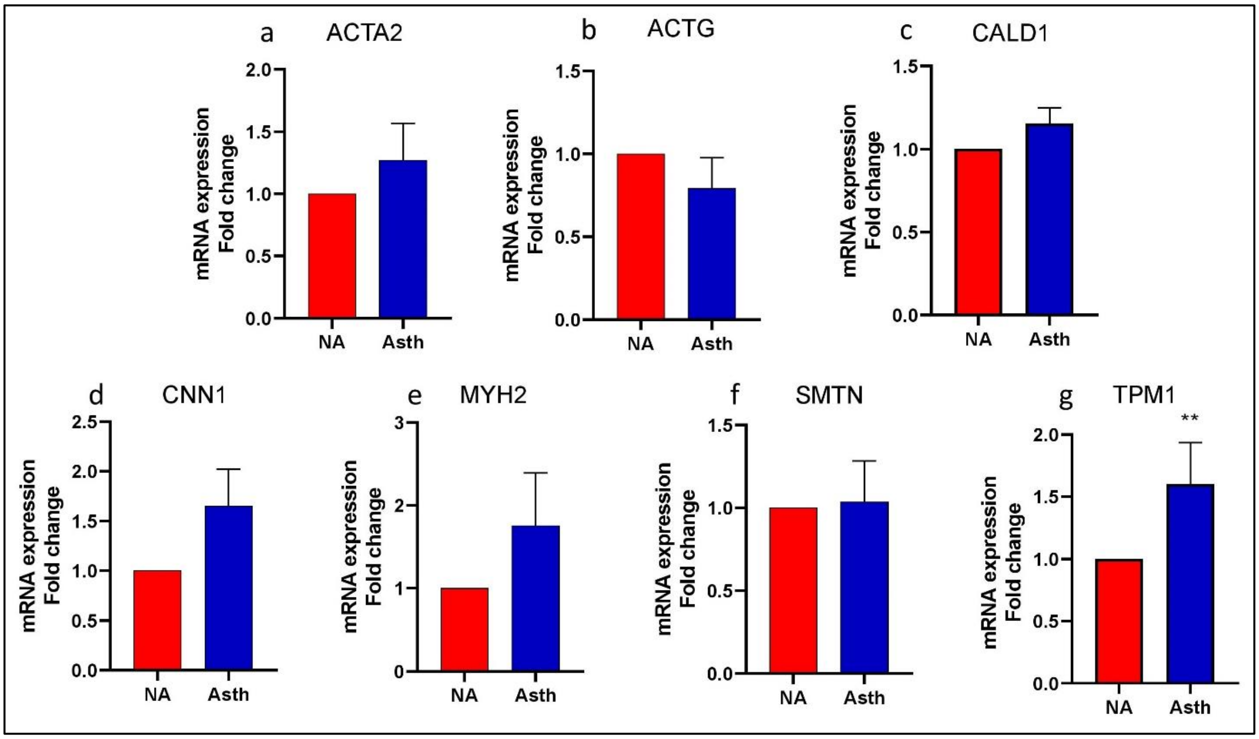

Supplementary Figure S1: Differential mRNA expression of smooth muscle markers in asthmatic and non-asthmatic human ASM samples. The fold change for mRNA was evaluated in both groups. The genes were validated in human ASM samples collected from non-asthmatic (NA) and asthmatic (Asth) patients (N = 5 in each group). Data represented as mean ± SEM in each group. **p-value<0.001.
